# Integrated Single‐Cell and Spatial Transcriptome Reveal Metabolic Gene *SLC16A3* as a Key Regulator of Immune Suppression in Hepatocellular Carcinoma

**DOI:** 10.1111/jcmm.70272

**Published:** 2024-12-10

**Authors:** Qianlong Kang, Xiaomeng Yin, Zhenru Wu, Aiping Zheng, Lusi Feng, Xuelei Ma, Li Li

**Affiliations:** ^1^ Department of Pathology and Institute of Clinical Pathology, West China Hospital Sichuan University Chengdu China; ^2^ Department of Biotherapy, Cancer Center and State Key Laboratory of Biotherapy, West China Hospital Sichuan University Chengdu China; ^3^ Frontiers Science Center for Disease‐Related Molecular Network, West China Hospital Sichuan University Chengdu China

**Keywords:** hepatocellular carcinoma, metabolic reprogramming, *SLC16A3*, spatial transcriptomics, tumour microenvironment

## Abstract

Hepatocellular carcinoma (HCC) is one of the most lethal cancers, usually diagnosed at an advanced stage. Metabolic reprogramming plays a significant role in HCC progression, probably related to immune evasion, yet the key gene is unclear. In this study, six metabolism‐related genes with prognostic implications were screened. Correlation analysis between the key genes and immune cell subtypes was conducted, and a prominent gene strongly associated with immunosuppression, *SLC16A3*, was identified. Overexpression of *SLC16A3* is associated with the loss of T‐cell function and might lead to the upregulation of several immunosuppressive proteins. Gene function enrichment analysis showed genes correlated with *SLC16A3* primarily involved in cell adhesion. Single‐cell analysis showed that the *SLC16A3* gene was mainly expressed in macrophages, especially some tumour‐promoting macrophages. Further analysis of spatial transcriptome data indicated that *SLC16A3* was enriched at the tumour invasion front. The mIHC revealed that patients with high *SLC16A3* expression exhibited significantly reduced infiltration of GZMB^+^ cells. And *SLC16A3* inhibitors significantly suppressed the proliferation of HCC, while simultaneously enhancing T‐cell cytotoxicity and reducing exhaustion. These results reveal the phenomenon of immune escape mediated by metabolic reprogramming and suggest that *SLC16A3* may serve as a novel target for intervention.

## 1 Introduction

Hepatocellular carcinoma (HCC) stands as the most prevalent form of primary liver cancer and ranks as the third leading cause of cancer‐related mortality globally. The relative 5‐year survival rate is around 18% [1]. Although surgical resection is the preferred therapeutic intervention for early‐stage HCC patients, the inevitability of high recurrence rates within 5 years persists [2]. Regrettably, a large proportion of HCC patients receive diagnoses at an advanced stage. Transcatheter arterial chemoembolization (TACE) and chemotherapy emerge as primary strategies for such patients, albeit with unsatisfactory prognoses [3]. In recent years, immunotherapy has emerged as a novel and promising approach for HCC treatment. The IMbrave 150 trial, in particular, demonstrated extended overall and progression‐free survival in unresectable HCC patients treated with atezolizumab plus bevacizumab compared to sorafenib [4]. However, randomised clinical trials (KEYNOTE‐240 and CheckMate‐459) of immune checkpoint inhibitor anti‐PD1 therapy in patients with HCC did not show statistically significant improvement. In addition, more PD‐1 ICB resistance was seen during immunotherapy in patients with hepatocellular carcinoma [5], the efficacy of immunotherapy remains uncertain, potentially attributed to suppressed tumour microenvironments [6, 7] and immune evasion [8].

Metabolic reprogramming plays a crucial role in tumour initiation and progression. Tumour cells exploit metabolic reprogramming to adopt distinct metabolic pathways compared to normal cells. These alterations include the upregulation of glycolysis, gluconeogenesis and β‐oxidation, enabling rapid proliferation and heightened energy metabolism [9]. Additionally, recent studies revealed metabolic reprogramming occurring within various components of the tumour microenvironment, notably immune cells [10]. Reprogramming in immune cell metabolism can lead to altered function of immune cells, ultimately fostering immune evasion of tumour cells. Targeting genes linked to metabolic reprogramming presents a novel way for therapeutic intervention to prevent immune evasion. *SLC16A3* (solute carrier family 16 member 3) belongs to the solute carrier 16 (SLC16) gene family, a group encompassing diverse monocarboxylate transporters (MCTs), which can transport lactic acid and pyruvate across plasma membranes [7, 11, 12]. High expression of *SLC16A3* has been related to elevated glycolytic metabolism in various cancers, which showed its potential as a prognostic marker associated with metabolic reprogramming of cancers [13, 14, 15]. In addition, *SLC16A3* might be associated with decreased CD8^+^ T‐cell infiltration and increased myeloid‐derived suppressor cells (MDSC) enrichment, indicating that targeting *SLC16A3* may reverse the suppressive tumour microenvironment and enhance the efficacy of anti‐PD‐1 therapy [11, 15, 16].

Single‐cell sequencing has been widely applied in the field of Oncology, characterising the genetic, epigenetic and transcriptional heterogeneity within tumours at a single‐cell resolution and helping understand the tumour microenvironment [17]. Its role in dissecting metabolic reprogramming of cancer is also gradually revealed, as it can detect the metabolites of single cells and help identify the relationship between metabolism and specific cell population [18, 19, 20]. However, the spatial heterogeneity of tumour tissues stands as a pivotal characteristic, representing a key feature of tumours. The positional information of various cells in the tumour microenvironment holds significant importance in tumour lineage development [21]. Therefore, to achieve a more comprehensive understanding of tumours, it is necessary to analyse both their transcriptional information and spatial coordinates.

In the current study, we applied a comprehensive approach that correlates metabolic gene analysis with immune cells based on gene expression profiles of HCC patients collected from The Cancer Genome Atlas (TCGA) database. We identified a metabolic‐related gene, *SLC16A3*, that significantly influences the tumour microenvironment. Integrating scRNA‐seq and spatial transcriptomics data revealed that *SLC16A3* is predominantly expressed in macrophages and is associated with reduced T‐cell cytotoxicity and increased exhaustion. We found *SLC16A3* inhibitors can significantly suppress HCC proliferation, while enhancing T‐cell cytotoxicity and reducing exhaustion. Our study unveiled a metabolic‐related immune evasion in HCC and suggested *SLC16A3* as a potential therapeutic target.

## 2 Materials and Methods

### 2.1 Data Acquisition

We initially acquired the HCC expression dataset, along with sample phenotype data, from the UCSC database (https://xena.ucsc.edu/), comprising a collection of 424 samples collected until July 19, 2019. This dataset encompasses both normal and HCC samples.

### 2.2 Identification of Differentially Expressed Genes (DEGs) Related to Metabolism

We conducted differential expression analysis on the entire transcriptome matrix using the R package edgeR. We conducted gene ontology (GO) and KEGG enrichment analyses on these genes with differential expression. Subsequently, we subsetted the FPKM dataset to include genes associated with metabolism and obtained a metabolism‐related transcriptome expression matrix. Leveraging the R package edgeR, we performed differential analysis to identify metabolism‐related DEGs. These genes were then subjected to visualisation analysis to elucidate their distribution within each metabolic pathway.

### 2.3 Analysis of Prognostic‐Related Gene

Building upon the foundation of DEGs, we acquired HCC survival data from UCSC, comprising 463 samples. We performed correlation analysis between survival data and differential expression data, primarily employing the Cox function from the R package survival to conduct univariate hazard analysis. This facilitated the identification of prognostic‐related genes and their respective hazard ratios (HR). By associating prognostic genes with metabolic gene sets, genes with Cox *p*‐values less than 0.01 are considered significant. Subsequently, we used the survfit function to construct Kaplan–Meier survival curves for these key genes.

### 2.4 Immune Profile Analysis

The ESTIMATE algorithm [22] was used to evaluate the stromal score, immune score and tumour purity. The CIBERSORT algorithms were used to infer the proportion of immune cells in the tumour microenvironment. Further, we established correlations using Spearman method between key genes and immune cell types, analysing the degree of their association.

### 2.5 Correlation Analysis Between Macrophage and Key Genes

By correlating *SLC16A3* expression levels with upregulated and downregulated *CD68* and *CD8* genes, categorise these genes into four groups. We subsequently conducted prognostic analysis on these gene subgroups to assess their impact on liver cancer patient prognosis. Further, we performed a correlation analysis between each subgroup and immune factors to obtain their expression profiles within each immune factor category.

### 2.6 Functional Enrichment Analysis

To investigate the broader impact of *SLC16A3* on the tumour immune microenvironment, we correlated the expression gene matrix of liver cancer with both *SLC16A3* and *CD68*. This analysis yielded three significant subgroups, which were subsequently subjected to functional enrichment analysis to extract relevant functional information. The R package clusterProfiler was used to perform GO functional enrichment analysis [23].

### 2.7 scRNA‐Seq Data Analysis

We downloaded the single‐cell RNA transcription data of HCC from the GSE149614 [24] in the Gene Expression Omnibus (GEO) database, which included 34,414 cells. The raw count expression profiles were normalised and scaled using the Seurat (V4) R package and genes were removed if they expressed in < 0.1% of cells. The Top 2000 high variable features of the dataset were applied to perform a principal component analysis for reducing the dimensionality of data, and the top 30 principal components were used for downstream analyses. The Harmony algorithm with default parameters was employed to correct batch effects among samples. The cell subpopulations were identified using the ‘FindClusters’ function (resolution = 0.1) and visualised using the UMAP method. We utilised the ‘FindMarkers’ function to compare the differences between T cells in the two groups.

### 2.8 Spatial Transcriptomics Data Analysis

We collected spatial transcriptomics data of HCC from the previous study [25]. For each patient, the top 2000 high variable features were applied to perform a principal component analysis for reducing the dimensionality of the spot data, and the top 20 principal components were used for downstream analyses. Then, we performed unsupervised shared nearest neighbour (SNN) based clustering and UMAP visualisation analysis. Each cluster was annotated based on histomorphology and corresponding gene expression to identify the tumour, normal and transition regions. We used the geometric mean of *CD68* and *SLC16A3* expression to identify spots where *SLC16A3* ^+^ macrophages might be present.

### 2.9 Mice and In Vivo Experiments

Animal tumour experiments were conducted in accordance with the regulations of the Experimental Animal Ethics Committee of West China Hospital, Sichuan University and were approved by the same committee. To generate Hepa 1‐6 tumours in mice, 1 × 10^6^ Hepa 1‐6 tumour cells were suspended in 100 μL of DMEM medium and injected subcutaneously into the dorsal side of the mice. The dose of MCT4i (MCE #MSC‐4381) was 30 mg/kg body weight, and the MCT inhibitor was administered intraperitoneally daily starting on the 6th day after tumour inoculation. Tumour volume was determined by measuring the length and width of the tumours. At designated time points, mice were euthanized by cervical dislocation, and subcutaneous tumours were collected for flow cytometry analysis and paraffin embedding.

### 2.10 Multiplex Immunohistochemistry Staining

Tissue microarrays of hepatocellular carcinoma were obtained from the Department of Pathology, West China Hospital. Mouse tumour tissues were fixed with paraformaldehyde. Paraffin sections were baked at 65°C for 4 h, followed by dewaxing and rehydration using xylene and graded alcohol. For antigen retrieval, either EDTA buffer (pH 9.0) or sodium citrate buffer (pH 6.0) was employed. To minimise nonspecific antibody binding, the sections were preincubated with an antibody diluent. Sequential incubation with primary and secondary antibodies was carried out in accordance with the experimental protocol. Multiplex immunohistochemistry (mIHC) staining was performed using an Opal 7‐Color Automation IHC Kit (Akoya Biosciences, Marlborough, MA), following the manufacturer's guidelines. Following primary antibody incubation, the slides underwent secondary antibody incubation, followed by tyrosine signal amplification. Afterward, a new round of target incubation was conducted. The targets we detected included *SLC16A3* (Abcam ab308528, 1:100), CD68 (Cell Signaling Technology 76437, 1:200) and GZMB (Abcam ab255598, 1:3000). The immunohistochemical slides were analysed using the PhenoImager (Akoya Biosciences) and the results were quantified with QuPath software.

### 2.11 Flow Cytometry

Use scissors to cut the tumour tissue into small pieces. Digest the tissue in an enzyme mixture containing 2 mg/mL Collagenase II, 50 μg/mL DNase I, 2.5 mmol/L CaCl2 and 2% fetal bovine serum (FBS) diluted in RPMI 1640 medium. Incubate the mixture in a shaker at 37°C for 20 min. Filter the digested tissue through a 40 μm mesh to obtain a single‐cell suspension at a concentration of 1 × 10^6^ cells/mL. Incubate the cells with a viability dye (Invitrogen #65‐0866‐18) on ice in the dark for 30 min. Block the cells with 2% BSA in PBS on ice for 30 min. Stain the cells sequentially with extracellular antibodies (CD45 (Invitrogen #56‐0451‐82), CD3 (BioLegend # 100221), CD8 (Invitrogen #11‐0081‐81), CD11b (Invitrogen #406‐0112‐82), F4/80 (BioLegend # 123115), PD‐1 (Invitrogen #67‐9501‐82)) on ice in the dark for 30 min. Fix the cells with 4% paraformaldehyde for 20 min, then permeabilize them with 90% methanol according to the manufacturer's instructions. Finally, stain the cells with intracellular antibodies (CD206 (Invitrogen #12‐2061‐82), GZMB (Invitrogen #25‐8898‐82)).

### 2.12 Statistical Analysis

R (V 4.1.3) software was used for the above statistics and analysis. The images were stitched together by Adobe Illustrator software. Wilcoxon rank‐sum test was used for the comparison of box plots. The Spearman coefficient was used for correlation analysis. Kaplan–Meier method was used to plot the survival curve. Cox analysis was used to assess characteristics associated with survival. All hypothesis tests were two‐sided tests. *p*‐Values of multiple tests were corrected by the FDR method.

## 3 Results

### 3.1 Differential Expression Analysis of Metabolism‐Related Genes

**FIGURE 1:**
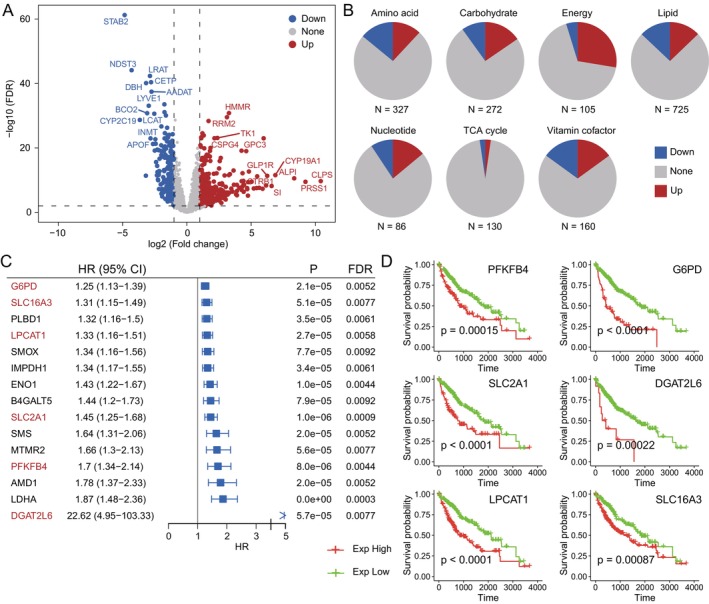
Correlation of metabolism‐related DEGs and prognosis. (A) Volcano plot of metabolism‐related DEGs. (B) Percentage of DEGs in each metabolic pathway. (C) Prognosis‐related genes, (FDR < 0.01), metabolically differentially expressed prognostic genes (labelled in red). (D) Metabolically differentially expressed prognostic genes related to the survival curves (*p* < 0.01).

Through an analysis of the entire gene expression profile, we gained a comprehensive overview of widespread differential expression (Table S1). This was followed by conducting enrichment analysis on the differentially expressed genes, revealing their primary involvement in key metabolic pathways and functions (Figure S1). Subsequently, we associated this data with metabolic genes to construct a metabolic‐associated expression matrix (Table S2). We performed differential expression analysis on this matrix, identifying DEGs related to metabolism (Figure 1A). Pie chart results exhibited the distribution of differential genes across various metabolic pathways (Figure 1B).

### 3.2 Survival Analysis of Differentially Expressed Metabolism‐Related Genes

Survival analysis yielded 15 genes significantly correlated with prognosis. Subsequent association of prognostic genes with differential metabolism‐related genes resulted in the identification of six key DEGs related to metabolism and prognosis: *G6PD*, *SLC16A3*, *LPCAT1*, *SLC2A1*, *PFKFB4*, *DGAT2L6* (Figure 1C). Overexpression of these genes was associated with increasing risk of poor prognosis for liver cancer patients. Further analysis and validation through Kaplan–Meier curves confirmed that overexpression of these six key genes might lead to poorer prognosis among liver cancer patients (*p* < 0.01) (Figure 1D).

### 3.3 Immune‐Related Findings of Key Genes

**FIGURE 2:**
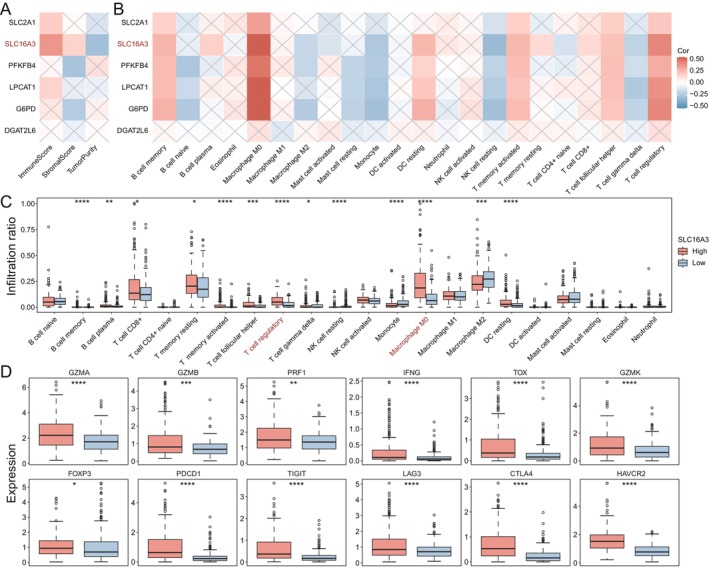
Impact of 6 key genes on immunity. (A, B) Immunity versus stroma scores of key genes and correlation with various types of immune cells. (C, D) Effects of overexpression and gene expression of *SLC16A3* on the expression of various immune cells as well as immune‐related factors. *P < 0.05; **P < 0.01; ***P < 0.001; ****P < 0.0001.

Previous studies have shown that metabolic reprogramming in cancers is related to immune response, including immune cell differentiation and functions [26, 27, 28]. Through our analysis of the correlation between the six metabolism‐related key genes and immune scores, stromal scores and tumour purity, it was revealed that within the liver cancer tumour microenvironment, *SLC16A3* exhibited a positive correlation with immune scores and the strongest negative correlation with tumour purity. This underscores the pivotal role of *SLC16A3* within the tumour microenvironment of liver cancer (Figure 2A). Subsequently, through further correlation analysis of these six key genes with various immune cell types, we found a pronounced positive correlation between *SLC16A3* and M0 macrophages (Figure 2B). This prompted us to spotlight *SLC16A3* for further analysis of its impact on tumour progression and treatment. Based on the median expression of *SLC16A3*, we categorised the patients into two groups. We observed that patients with high expression of *SLC16A3* tended to have a higher proportion of M0 macrophages and regulatory T‐cell infiltration, which were associated with immunosuppression (Figure 2C). Next, we examined the expression pattern of *SLC16A3* in various immune‐related factors (Figure 2D). Significant associations were observed between *SLC16A3* overexpression and upregulation of *TOX*, *PDCD1*, *TIGIT*, *CTLA4* and *HAVCR2* expression. Overall, our study identified a metabolic‐related gene, *SLC16A3*, which correlated with the tumour microenvironment, providing insights into the interplay between *SLC16A3* and the immune landscape within HCC.

### 3.4 Analysis of the Association Between *SLC16A3* and Immune Cells

**FIGURE 3:**
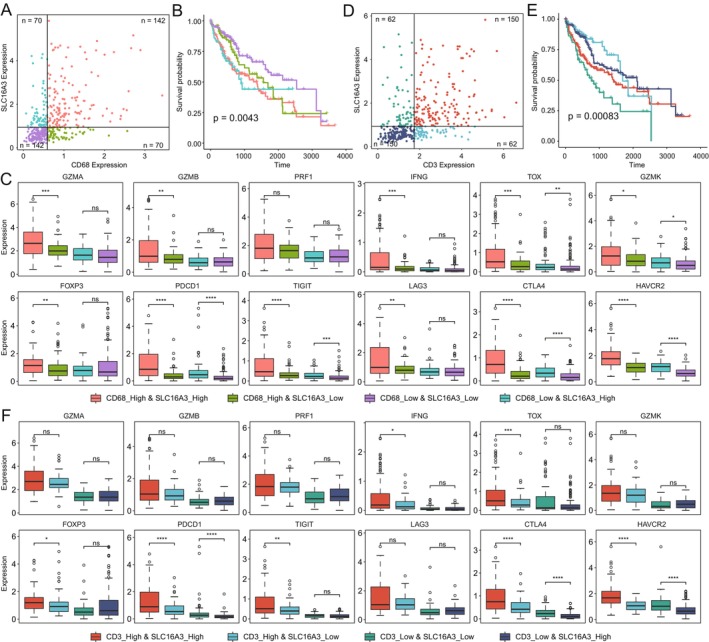
Correction of immune cell numbers' impact on *SLC16A3*‐mediated immune suppression. (A, B) The samples were categorised into four subgroups based on the expression levels of *SLC16A3* and *CD68*, and survival analysis of these subgroups yielded significant survival curves (*p* < 0.01). (C, D) Samples were divided into four subgroups based on the expression levels of *SLC16A3* and *CD3*, and subsequent survival analysis of these subgroups also revealed significant survival curves (*p* < 0.01).

We divided the samples into four subgroups based on the expression levels of *SLC16A3* and *CD68*: low expression of both *CD68* and *SLC16A3*, low expression of *CD68* and overexpression of *SLC16A3*, overexpression of *CD68* and low expression of *SLC16A3*, and overexpression of both *CD68* and *SLC16A3* (Figure 3A). Survival analysis was performed for each subgroup, revealing that the purple subgroup, characterised by low expression of both *CD68* and *SLC16A3*, exhibited the best prognosis, followed by the subgroup with overexpression of *CD68* and low expression of *SLC16A3* (Figure 3B). These survival curves indicated that overexpression of *SLC16A3* might lead to unfavourable prognosis in liver cancer patients. Subsequently, we conducted a comprehensive analysis of the expression levels of various immune factors within these four subgroups (Figure 3C). The box plots clearly illustrated significant differences in the expression levels of *GZMA*, *GZMB*, *IFNG*, *TOX*, *FOXP3*, *PDCD1*, *TIGIT*, *LAG3*, *CTLA4* and *HAVCR2*, regulated by *SLC16A3* expression, particularly in scenarios with a higher number of macrophages. Notably, even when the macrophage count was lower, significant differences in the expression of *TOX*, *PDCD1*, *TIGIT*, *CTLA4* and *HAVCR2* still existed between the upregulated and downregulated *SLC16A3* groups, indicating that the inhibitory status induced by *SLC16A3* overexpression persisted after adjusting for macrophage quantity. Subsequently, using a similar approach based on the expression levels of *SLC16A3* and *CD3*, we subdivided the samples into four groups (Figure 3D,E). The results indicated that despite variations in the number of T cells, the upregulation and downregulation of *SLC16A3* still significantly associated with the regulation *PDCD1*, *CTLA4* and *HAVCR2* expression, implying a notable impact on immune suppression (Figure 3F). These findings suggested that the immune inhibitory effects induced by *SLC16A3* persisted even after correcting for the quantity of T cells. To further validate the relationship between *SLC16A3*, macrophage count and T‐cell quantity, we chose one of these directions as our main research focus and further investigated the association between *SLC16A3* and macrophage count.

### 3.5 *SLC16A3* and Macrophage Subgroup‐Related Pathways

**FIGURE 4:**
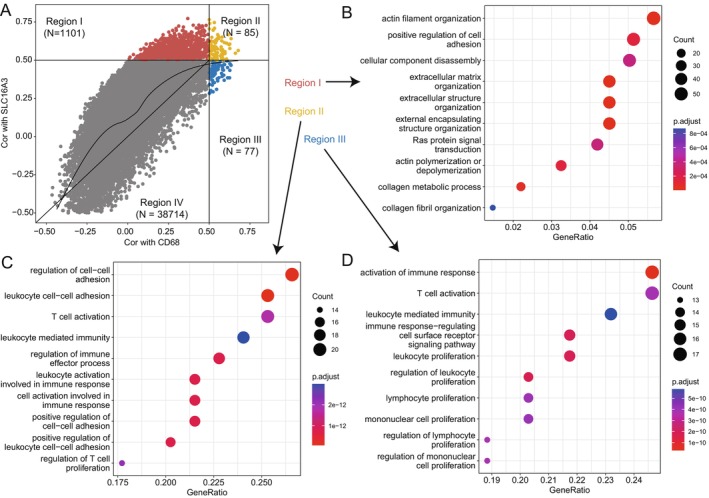
Gene function enrichment analysis of *SLC16A3*‐related genes. (A) Association of all genes with *SLC16A3* and *CD68* to obtain three major gene clusters; (B–D) Functional enrichment of genes in each cluster.

To elucidate the mechanisms behind *SLC16A3*‐mediated immune suppression, we subsequently conducted an association analysis between all genes and *CD68* as well as *SLC16A3*. We identified genes that were correlated with *SLC16A3* and macrophages and classified them into four subgroups based on their correlations (Figure 4A). These subgroups included genes that were highly positively correlated with *SLC16A3* and negatively correlated with *CD68* (Region I); genes highly positively correlated with both *SLC16A3* and *CD68* (Region II); genes highly positively correlated with *CD68* and negatively correlated with *SLC16A3* (Region III); and genes with low correlations to both *SLC16A3* and *CD68* (Region IV). Our primary focus was on the first three subgroups. Functional enrichment analysis revealed that genes in Region I were mainly associated with actin filament organisation, positive regulation of cell adhesion and cellular component disassembly (Figure 4B). These metabolic pathways primarily contributed to cell adhesion and degradation. Positive regulation of cell adhesion can effectively promote tumour metastasis progression [29]. Cancer cell metastasis is an extremely complex dynamic process that spans multiple spatiotemporal scales and involves various proteins at the molecular level based on force‐chemogenesis coupling interactions, including cytoskeleton polymerisation, assembly, depolymerization and remodelling mechanisms. Thus, the enrichment of the actin filament pathway further indicated that genetic alterations played a role in the mechanism of tumour metastasis [30]. Genes in Region II were enriched in pathways related to the regulation of cell–cell adhesion, leukocyte cell–cell adhesion, T‐cell activation and leukocyte‐mediated immunity (Figure 4C). The enrichment of these pathways indicated that the innate immune mechanism was activated within the tumour, effectively activating T‐cell immune killing and thereby inhibiting tumour progression. In Region III, genes were primarily associated with the activation of immune responses, T‐cell activation and leukocyte‐mediated immunity (Figure 4D). The enrichment results of the entire pathway showed that the immune response was activated, effectively promoting the anti‐tumour response. This indicated that under conditions of low *SLC16A3* expression, the liver cancer microenvironment had the potential for enhancing immune response activation. These findings collectively suggested that the interplay between *SLC16A3* and macrophages was intricately linked to the modulation of pathways related to cell adhesion, immune response activation and immune cell‐mediated functions in the liver cancer microenvironment.

### 3.6 Expression of *SLC16A3* Correlates With T‐Cell Dysfunction

**FIGURE 5:**
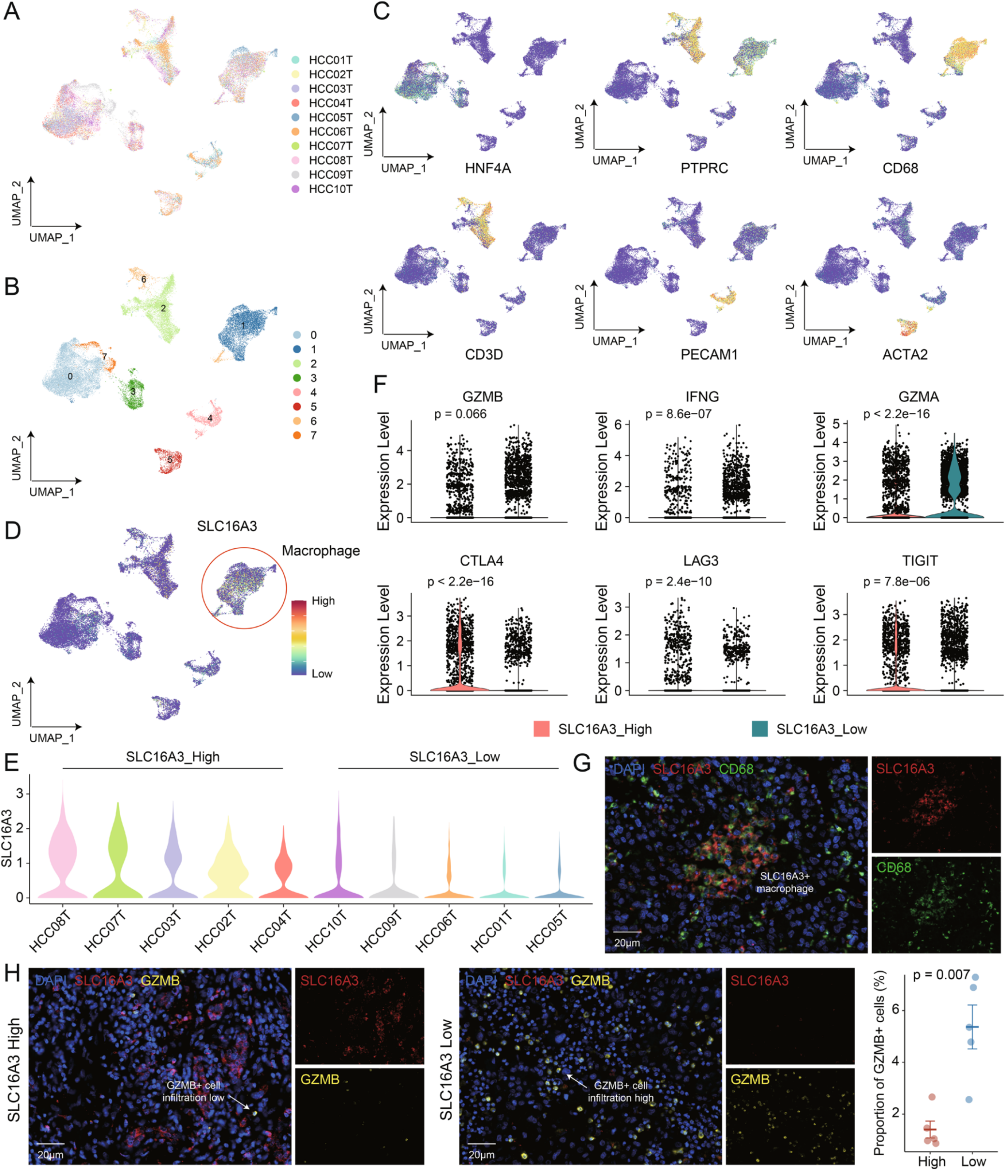
Deciphering the impact of *SLC16A3* on the immune microenvironment through Single‐Cell Data. (A) Umap containing 10 different samples. (B) Umap of 8 cell clusters. (C) Umap of each cluster marker. (D) Umap of *SLC16A3* expression. (E) The violin plot shows the distribution of *SLC16A3* expression in each sample. (F) The violin plot illustrates differences in the expression of molecules in T cells between patient groups with high and low *SLC16A3* expression levels. (G) The mIHC staining demonstrates the macrophage and *SLC16A3* expression in liver cancer tissue. (H) Infiltration of SCL16A3^+^ and GZMB^+^ cells are visualised by mIHC staining in liver cancer tissue microarray. Quantifications of cell proportion are shown on the right. *N* = 5. Data are presented as mean ± SEM.

To investigate the role of *SLC16A3* in the immune microenvironment, we collected additional single‐cell data from 10 primary liver cancer patients (Figure 5A). After clustering and dimensionality reduction, a total of 8 cell subtypes were identified (Figure 5B). Based on classical cell markers, we identified hepatocytes (Cluster 0, 3, 7), macrophages (Cluster 1), T cells (Cluster 2, 6), endothelial cells (Cluster 4) and smooth muscle cells (Cluster 5) (Figure 5C). Interestingly, we observed prominent expression of *SLC16A3* primarily within macrophages (Figure 5D). Subsequently, based on the expression levels of *SLC16A3* in macrophages, we categorised the 10 samples into two groups: *SLC16A3*_high and *SLC16A3*_low (Figure 5E). We then compared the functional status of T cells between these two groups. The results demonstrated a substantial decrease in the expression of cytotoxic molecules like *GZMB*, *GZMA* and *IFNG* by T cells from *SLC16A3*_high samples (Figure 5F). Conversely, there was elevated expression of certain immune checkpoint molecules like *CTLA4*, *LAG3* and *TIGIT* (Figure 5F). IHC experiments confirmed the presence of macrophages expressing *SLC16A3* (Figure 5G). Meanwhile, in regions with high expression of *SLC16A3*, there was a significant decrease in the infiltration proportion of GZMB^+^ cells (Figure 5H).

**FIGURE 6:**
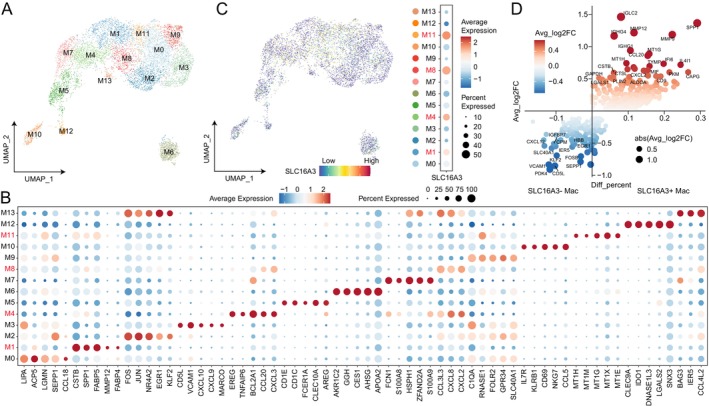
Expression Analysis of *SLC16A3* in Different Macrophage States. (A) Umap view of different macrophage clusters. (B) Expression levels and frequencies of selected markers across macrophage clusters. (C) The expression of *SLC16A3* is projected on the Umap plot of macrophages. The dotplot shows expression levels and frequencies of *SLC16A3* across macrophage clusters. (D) Scatter plot showing differential genes of *SLC16A3* ^+^ and *SLC16A3* ^−^ macrophages. Genes with corrected *p*‐values less than 0.01 in the differential analysis are displayed in the plot. The *x*‐axis represents the difference in gene expression proportions between the two cell groups, while the *y*‐axis represents the fold difference in gene expression means between the two cell groups.

Considering the diversity of macrophage states, we analysed the expression of *SLC16A3* among different macrophage states. Further clustering of macrophages resulted in 14 cellular subtypes (Figure 6A,B). We observed that *SLC16A3* was predominantly expressed at high levels in M11, M8, M4 and M1 (Figure 6C). Among these, M11 exhibited high expression of metallothionein, and M4 expressed *EREG*, which promotes tumour growth [31]. M1 was characterised by high expression of *SPP1*, and previous studies have associated this with an adverse prognosis [24]. Differential analysis indicated that *SLC16A3* ^+^ macrophages exhibit high expression of macrophage markers promoting migration, invasion and tumour angiogenesis, such as *SPP1* and *MMP9*, as well as genes associated with glycolysis, including *PKM* (Figure 6D) [24, 32, 33, 34, 35]. Collectively, these findings suggested that tumour‐promoting macrophage expression of *SLC16A3* might lead to impaired T‐cell functionality, consequently contributing to the establishment of an immunosuppressive microenvironment.

### 3.7 *SLC16A3* ^+^ Macrophages Were Enriched at the Front of Tumour Invasion

**FIGURE 7:**
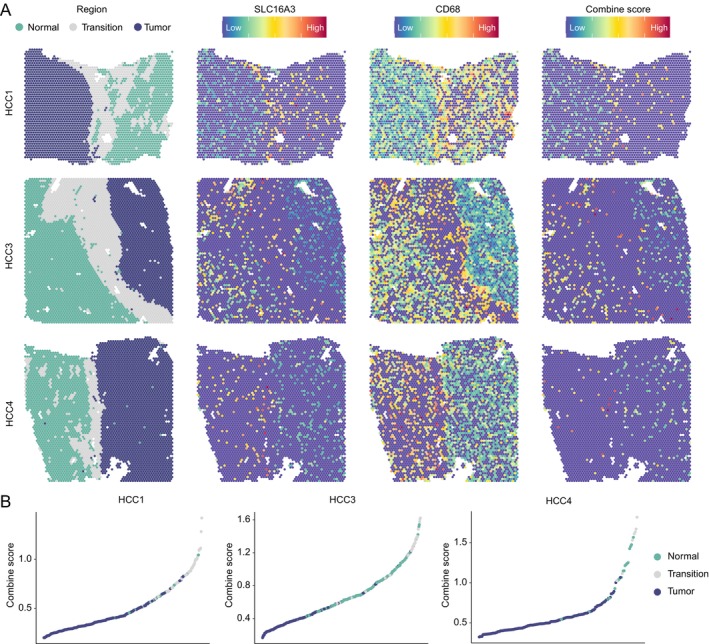
Spatial distribution of *SLC16A3* ^+^ macrophages. (A) The normal, tumour and transition region distribution and gene expression of each section. (B) Scatter plots show the distribution of *SLC16A3* ^+^ macrophage scores across regions of each section.

Next, we examined the spatial distribution characteristics of *SLC16A3* ^+^ macrophages. We collected spatial transcriptome data from three samples, each containing tumour and normal regions, from previously published articles [25]. After performing dimensionality reduction clustering for each sample's spots, these three samples exhibited typical transitional zones between normal and tumour regions (Figure S2). Subsequently, we analysed the spatial expression of *SLC16A3* and *CD68* in these regions, using the geometric mean of both to characterise the distribution characteristics of *SLC16A3* ^+^ macrophages (Figure 7A). The results indicated that these *SLC16A3* ^+^ macrophages were primarily concentrated in the transitional zone at the tumour front and some distribution in the normal area before the transitional zone (Figure 7B), suggesting that *SLC16A3* ^+^ macrophages might create conditions for tumour cell expansion and invasive growth [36].

### 3.8 Inhibition of *SLC16A3* Suppress the Proliferation of HCC by Enhancing Immunity

**FIGURE 8:**
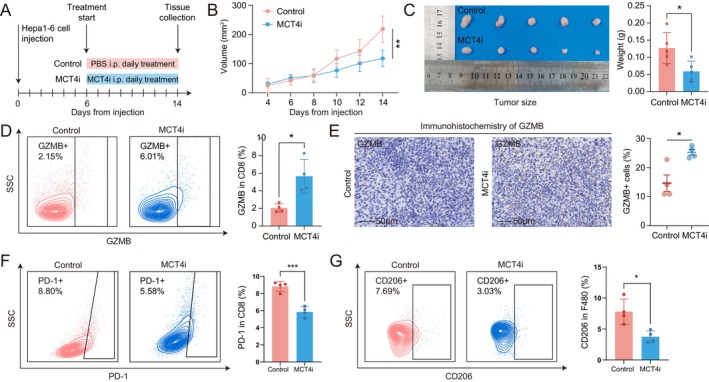
In vivo experiments validated the impact of *SLC16A3* on tumour progression. (A) Timeline of the mouse tumour model treatment. (B) Growth curve of subcutaneous tumour volume from tumour inoculation to euthanasia. (C) Images of subcutaneous hepatocellular carcinoma tumours in mice and tumour mass. (D) Flow cytometry analysis of GZMB expression. (E) Immunohistochemistry analysis of GZMB expression. (F) Flow cytometry analysis of PD‐1 expression in tumour tissues. (G) Flow cytometry analysis of macrophage subtypes in the tumour. *P < 0.05; **P < 0.01; ***P < 0.001.

We established a subcutaneous tumour model of mouse hepatocellular carcinoma (HCC) and utilised an *SLC16A3* inhibitor (MCT4i) to investigate the role of *SLC16A3* in HCC progression (Figure 8A). Results showed that the tumour volume growth rate of mice injected with MCT4i was significantly lower than that of the control group (Figure 8B). Weighing the subcutaneous tumours obtained revealed that the tumour mass in the MCT4i group was significantly lower than that in the control group (Figure 8C). Detection of the tumour GZMB marker by flow cytometry and immunohistochemistry methods, respectively, showed a significant upregulation of GZMB expression in the MCT4i group (Figure 8D,E), indicating that inhibiting *SLC16A3* could effectively enhance the cytotoxic ability of immune cells. Finally, flow cytometry results showed a significant decrease in PD‐1 expression of CD8 cells in the MCT4i group (Figure 8F), indicating that inhibiting the *SLC16A3* target could effectively reduce T‐cell exhaustion. Additionally, we found a significant decrease in CD206^+^ macrophage in the MCT4i group (Figure 8G). These results indicate that inhibiting *SLC16A3* can create a favourable immune microenvironment that slows the proliferation of hepatocellular carcinoma.

## 4 Discussion

Historically, the primary approaches for treating HCC have revolved around surgical resection and liver transplantation, while the widespread adoption of immune transplantation remains elusive [37]. Although radiotherapy and chemotherapy have been utilised to address HCC [38], these interventions impose significant psychological burdens on patients. However, individual variations persist, highlighting the need for a deeper comprehension of the immune landscape within HCC tumours to enable more precise treatment strategies. In this study, we identified six metabolism‐related prognostic genes in HCC and conducted comprehensive correlation analyses between these key genes and various immune cell types. As a result, we identified *SLC16A3* as a pivotal gene highly associated with immunity. Integrating single‐cell transcriptomics and spatial transcriptomics data revealed that *SLC16A3* is primarily expressed in macrophages and is associated with reduced T‐cell cytotoxicity. Further studies demonstrated that *SLC16A3* inhibitors significantly suppress HCC proliferation while enhancing T‐cell cytotoxicity and reducing exhaustion. These findings suggest that *SLC16A3* is a potential therapeutic target for liver cancer.

*SLC16A3* has a higher affinity for lactate than pyruvate, making it a significant lactate transporter [39]. High *SLC16A3* expression tend to regulate transportation of lactate to the extracellular environment, which may alter the pH level in the microenvironment [40, 41]. Notably, previous study has linked the lactate transporter *SLC16A3* to the tumour immune microenvironment, particularly in breast cancer. Previous studies usually evaluated the expression of *SLC16A3* in the entire cancer tissue and considered its role in the overall tumour environment. Earlier research has explored the crucial role of *SLC16A3* overexpression in tumour progression [42]. The presence of lactate has been shown to impede the polarisation process of M1 macrophages [43]. Upon *SLC16A3* expression in macrophages, the transporter facilitates the export of lactate from cells, resulting in a decrease in tumour microenvironment pH. This extracellular acidosis contributes to cancer progression [44]. Multiple studies have reported that a low pH environment can lead to impaired functionality of CD8^+^ T cells and reduced cytokine secretion, potentially attributed to mTORC1 suppression [44, 45, 46]. Additionally, extracellular acidification inhibits NK cell activity and elevates M2 macrophage levels, which are associated with a poorer prognosis [47, 48]. Lactate has also been implicated in the inhibition of M1 macrophage marker expression [43]. Therefore, it could be hypothesized that *SLC16A3*, expressed by macrophages, transports lactate extracellularly, inducing an acidotic immune microenvironment that suppresses T‐cell function, facilitates immune escape and contributes to an unfavourable prognosis.

Our analysis, which correlated *SLC16A3* overexpression with the expression levels of diverse immune factors, revealed upregulation of *TOX*, *PDCD1*, *TIGIT*, *CTLA4* and *HAVCR2*. Notably, elevated *TOX* expression triggers T‐cell exhaustion, thereby promoting immune suppression [49]. *PDCD1* encodes programmed cell death protein 1, a cell surface protein that hinders immune responses against human cells [50]. On the other hand, *TIGIT* functions as an immunosuppressive cell surface protein, and *CTLA4* encodes a protein that transmits inhibitory signals to T cells, exerting immunosuppresion [51, 52]. Previous research has demonstrated that knocking out the *HAVCR2* gene in dendritic cells significantly inhibits tumour growth and notably increases CD8^+^ T‐cell infiltration in tumours, encompassing early activated, effector, memory and memory precursor T cells, all of which play a crucial role in anti‐tumour immune responses [53]. Hence, modulation of these immune factors could potentially explain a mechanism through which *SLC16A3* contributes to tumour microenvironment suppression.

However, it is important to acknowledge the limitations of this study. First, all analyses were based on data from public databases, potentially introducing bias. Future investigations should focus on exploring underlying mechanisms to uncover the pivotal role of *SLC16A3* in regulating the metabolic reprogramming of the tumour microenvironment.

## 5 Conclusion

Overall, our study identified a metabolic‐related gene, *SLC16A3*, which correlates with the tumour microenvironment and validated its immunosuppressive function in the tumour microenvironment through in vivo experiments, providing insights into the interplay between *SLC16A3* and the immune landscape within HCC. *SLC16A3* expression by pro‐tumour macrophages may be related to T‐cell dysfunction and tumour invasion. Our findings suggested potential therapeutic strategies aimed at harnessing *SLC16A3*'s impact on the tumour microenvironment to develop more effective treatment approaches for HCC patients and improve their prognosis.

## Author Contributions

**Qianlong Kang:** data curation (equal), formal analysis (equal), methodology (equal), project administration (equal), resources (equal), software (equal), validation (equal), visualization (equal), writing – original draft (equal), writing – review and editing (equal). **Xiaomeng Yin:** conceptualization (equal), data curation (equal), investigation (equal), methodology (equal), project administration (equal), resources (equal), software (equal), validation (equal), visualization (equal), writing – original draft (equal), writing – review and editing (equal). **Zhenru Wu:** conceptualization (equal), formal analysis (equal), funding acquisition (equal), methodology (equal), writing – original draft (equal). **Aiping Zheng:** conceptualization (equal), data curation (equal), formal analysis (equal), methodology (equal), project administration (equal), resources (equal), software (equal), validation (equal), visualization (equal), writing – original draft (equal), writing – review and editing (equal). **Lusi Feng:** conceptualization (equal), data curation (equal), formal analysis (equal), methodology (equal), project administration (equal), resources (equal), software (equal), validation (equal), visualization (equal), writing – original draft (equal), writing – review and editing (equal). **Xuelei Ma:** conceptualization (lead), project administration (lead), supervision (lead), writing – review and editing (lead). **Li Li:** conceptualization (lead), supervision (lead), writing – review and editing (lead).

## Conflicts of Interest

The authors declare that they have no conflicts of interest. Part of the figures were created with biorender.com.

## Supporting information


**Figure S1.** Hepatocellular carcinoma public database transcriptome differential analysis. (A) Differential gene expression volcano plot. (B) Gene Ontology (GO) enrichment analysis of differentially expressed genes. (C) KEGG enrichment analysis of differentially expressed genes.

**Figure S2.** Spatial transcriptome data of three samples. H&E staining (A), spatial cluster distribution (B) and spatial feature plots of COL1A1 expression (C) of each section.

**Table S1.** Differentially expressed gene set in hepatocellular carcinoma, Related to Figure S1.

**Table S2.** The gene sets of the seven metabolic super pathways surveyed, Related to Figure 1.

## Data Availability

All data are from the UCSC database (https://xena.ucsc.edu/). The computer code used in this study, mIHC image and other materials related with this study were available on reasonable request.

## References

[1] A. Villanueva , “Hepatocellular Carcinoma,” New England Journal of Medicine 380, no. 15 (2019): 1450–1462, 10.1056/NEJMra1713263. 30970190

[2] T. Ishizawa , K. Hasegawa , T. Aoki , et al., “Neither Multiple Tumors nor Portal Hypertension Are Surgical Contraindications for Hepatocellular Carcinoma,” Gastroenterology 134, no. 7 (2008): 1908–1916, 10.1053/j.gastro.2008.02.091. 18549877

[3] J. M. Llovet , F. Castet , M. Heikenwalder , et al., “Immunotherapies for Hepatocellular Carcinoma,” Nature Reviews Clinical Oncology 19, no. 3 (2022): 151–172, 10.1038/s41571-021-00573-2. 34764464

[4] R. S. Finn , S. Qin , M. Ikeda , et al., “Atezolizumab plus Bevacizumab in Unresectable Hepatocellular Carcinoma,” New England Journal of Medicine 382, no. 20 (2020): 1894–1905, 10.1056/NEJMoa1915745. 32402160

[5] Z. Tan , M. S. Chiu , X. Yang , et al., “Isoformic PD‐1‐Mediated Immunosuppression Underlies Resistance to PD‐1 Blockade in Hepatocellular Carcinoma Patients,” Gut 72, no. 8 (2023): 1568–1580, 10.1136/gutjnl-2022-327133. 36450387

[6] Z. Wang and X. Wu , “Study and Analysis of Antitumor Resistance Mechanism of PD1/PD‐L1 Immune Checkpoint Blocker,” Cancer Medicine 9, no. 21 (2020): 8086–8121, 10.1002/cam4.3410. 32875727 PMC7643687

[7] D. Vijayan , A. Young , M. W. L. Teng , and M. J. Smyth , “Targeting Immunosuppressive Adenosine in Cancer,” Nature Reviews Cancer 17, no. 12 (2017): 709–724, 10.1038/nrc.2017.86. 29059149

[8] R. S. Finn , B. Y. Ryoo , P. Merle , et al., “Pembrolizumab as Second‐Line Therapy in Patients With Advanced Hepatocellular Carcinoma in KEYNOTE‐240: A Randomized, Double‐Blind, Phase III Trial,” Journal of Clinical Oncology: Official Journal of the American Society of Clinical Oncology 38, no. 3 (2020): 193–202, 10.1200/jco.19.01307. 31790344

[9] X. Li , P. Ramadori , D. Pfister , M. Seehawer , L. Zender , and M. Heikenwalder , “The Immunological and Metabolic Landscape in Primary and Metastatic Liver Cancer,” Nature Reviews Cancer 21, no. 9 (2021): 541–557, 10.1038/s41568-021-00383-9. 34326518

[10] I. Martínez‐Reyes and N. S. Chandel , “Cancer Metabolism: Looking Forward,” Nature Reviews Cancer 21, no. 10 (2021): 669–680, 10.1038/s41568-021-00378-6. 34272515

[11] T. Yu , Z. Liu , Q. Tao , et al., “Targeting Tumor‐Intrinsic *SLC16A3* to Enhance Anti‐PD‐1 Efficacy via Tumor Immune Microenvironment Reprogramming,” Cancer Letters 589 (2024): 216824, 10.1016/j.canlet.2024.216824. 38522774

[12] P. D. Bosshart , R. P. Charles , R. A. Garibsingh , A. Schlessinger , and D. Fotiadis , “SLC16 Family: From Atomic Structure to Human Disease,” Trends in Biochemical Sciences 46, no. 1 (2021): 28–40, 10.1016/j.tibs.2020.07.005. 32828650

[13] G. Baek , Y. F. Tse , Z. Hu , et al., “MCT4 Defines a Glycolytic Subtype of Pancreatic Cancer With Poor Prognosis and Unique Metabolic Dependencies,” Cell Reports 9, no. 6 (2014): 2233–2249, 10.1016/j.celrep.2014.11.025. 25497091

[14] H. K. Kim , I. Lee , H. Bang , et al., “MCT4 Expression Is a Potential Therapeutic Target in Colorectal Cancer With Peritoneal Carcinomatosis,” Molecular Cancer Therapeutics 17, no. 4 (2018): 838–848, 10.1158/1535-7163.Mct-17-0535. 29483215

[15] L. Dong , D. Lu , R. Chen , et al., “Proteogenomic Characterization Identifies Clinically Relevant Subgroups of Intrahepatic Cholangiocarcinoma,” Cancer Cell 40, no. 1 (2022): 70–87 e15, 10.1016/j.ccell.2021.12.006. 34971568

[16] N. Li , Y. Kang , L. Wang , et al., “ALKBH5 Regulates Anti‐PD‐1 Therapy Response by Modulating Lactate and Suppressive Immune Cell Accumulation in Tumor Microenvironment,” Proceedings of the National Academy of Sciences of the United States of America 117, no. 33 (2020): 20159–20170, 10.1073/pnas.1918986117. 32747553 PMC7443867

[17] S. M. Lewis , M. L. Asselin‐Labat , Q. Nguyen , et al., “Spatial Omics and Multiplexed Imaging to Explore Cancer Biology,” Nature Methods 18, no. 9 (2021): 997–1012, 10.1038/s41592-021-01203-6. 34341583

[18] D. Wei , M. Xu , Z. Wang , and J. Tong , “The Development of Single‐Cell Metabolism and Its Role in Studying Cancer Emergent Properties,” Frontiers in Oncology 11 (2021): 814085, 10.3389/fonc.2021.814085. 35083160 PMC8784738

[19] L. Huang , H. Li , C. Zhang , et al., “Unlocking the Potential of T‐Cell Metabolism Reprogramming: Advancing Single‐Cell Approaches for Precision Immunotherapy in Tumour Immunity,” Clinical and Translational Medicine 14, no. 3 (2024): e1620, 10.1002/ctm2.1620. 38468489 PMC10928360

[20] D. Lambrechts , E. Wauters , B. Boeckx , et al., “Phenotype Molding of Stromal Cells in the Lung Tumor Microenvironment,” Nature Medicine 24, no. 8 (2018): 1277–1289, 10.1038/s41591-018-0096-5. 29988129

[21] E. Papalexi and R. Satija , “Single‐Cell RNA Sequencing to Explore Immune Cell Heterogeneity,” Nature Reviews Immunology 18, no. 1 (2018): 35–45, 10.1038/nri.2017.76. 28787399

[22] K. Yoshihara , M. Shahmoradgoli , E. Martínez , et al., “Inferring Tumour Purity and Stromal and Immune Cell Admixture From Expression Data,” Nature Communications 4 (2013): 2612, 10.1038/ncomms3612. PMC3826632 24113773

[23] T. Wu , E. Hu , S. Xu , et al., “clusterProfiler 4.0: A Universal Enrichment Tool for Interpreting Omics Data,” Innovation (Cambridge (Mass)) 2, no. 3 (2021): 100141, 10.1016/j.xinn.2021.100141. 34557778 PMC8454663

[24] Y. Lu , A. Yang , C. Quan , et al., “A Single‐Cell Atlas of the Multicellular Ecosystem of Primary and Metastatic Hepatocellular Carcinoma,” Nature Communications 13, no. 1 (2022): 4594, 10.1038/s41467-022-32283-3. PMC9357016 35933472

[25] R. Wu , W. Guo , X. Qiu , et al., “Comprehensive Analysis of Spatial Architecture in Primary Liver Cancer,” Science Advances 7, no. 51 (2021): eabg3750, 10.1126/sciadv.abg3750. 34919432 PMC8683021

[26] B. Chen , A. Gao , B. Tu , et al., “Metabolic Modulation via mTOR Pathway and Anti‐Angiogenesis Remodels Tumor Microenvironment Using PD‐L1‐Targeting Codelivery,” Biomaterials 255 (2020): 120187, 10.1016/j.biomaterials.2020.120187. 32590192

[27] B. Huang , B. L. Song , and C. Xu , “Cholesterol Metabolism in Cancer: Mechanisms and Therapeutic Opportunities,” Nature Metabolism 2, no. 2 (2020): 132–141, 10.1038/s42255-020-0174-0. 32694690

[28] L. Xia , L. Oyang , J. Lin , et al., “The Cancer Metabolic Reprogramming and Immune Response,” Molecular Cancer 20, no. 1 (2021): 28, 10.1186/s12943-021-01316-8. 33546704 PMC7863491

[29] C. Xu , K. Yang , Z. Xuan , et al., “BCKDK Regulates Breast Cancer Cell Adhesion and Tumor Metastasis by Inhibiting TRIM21 Ubiquitinate talin1,” Cell Death & Disease 14, no. 7 (2023): 445, 10.1038/s41419-023-05944-4. 37460470 PMC10352378

[30] X. Chen , Y. Li , M. Guo , et al., “Polymerization Force‐Regulated Actin Filament‐Arp2/3 Complex Interaction Dominates Self‐Adaptive Cell Migrations,” Proceedings of the National Academy of Sciences of the United States of America 120, no. 36 (2023): e2306512120, 10.1073/pnas.2306512120. 37639611 PMC10483647

[31] W. L. Cheng , P. H. Feng , K. Y. Lee , et al., “The Role of EREG/EGFR Pathway in Tumor Progression,” International Journal of Molecular Sciences 22, no. 23 (2021): 12828, 10.3390/ijms222312828. 34884633 PMC8657471

[32] J. Qi , H. Sun , Y. Zhang , et al., “Single‐Cell and Spatial Analysis Reveal Interaction of FAP(+) Fibroblasts and SPP1(+) Macrophages in Colorectal Cancer,” Nature Communications 13, no. 1 (2022): 1742, 10.1038/s41467-022-29366-6. PMC8976074 35365629

[33] J. Wei , Z. Chen , M. Hu , et al., “Characterizing Intercellular Communication of Pan‐Cancer Reveals SPP1^+^ Tumor‐Associated Macrophage Expanded in Hypoxia and Promoting Cancer Malignancy Through Single‐Cell RNA‐Seq Data,” Frontiers in Cell and Development Biology 9 (2021): 749210, 10.3389/fcell.2021.749210. PMC8523849 34676217

[34] A. X. Chen , R. D. Gartrell , J. Zhao , et al., “Single‐Cell Characterization of Macrophages in Glioblastoma Reveals MARCO as a Mesenchymal Pro‐Tumor Marker,” Genome Medicine 13, no. 1 (2021): 88, 10.1186/s13073-021-00906-x. 34011400 PMC8136167

[35] Y. Liu , Z. Xun , K. Ma , et al., “Identification of a Tumour Immune Barrier in the HCC Microenvironment That Determines the Efficacy of Immunotherapy,” Journal of Hepatology 78, no. 4 (2023): 770–782, 10.1016/j.jhep.2023.01.011. 36708811

[36] L. Wu , J. Yan , Y. Bai , et al., “An Invasive Zone in Human Liver Cancer Identified by Stereo‐Seq Promotes Hepatocyte‐Tumor Cell Crosstalk, Local Immunosuppression and Tumor Progression,” Cell Research 33, no. 8 (2023): 585–603, 10.1038/s41422-023-00831-1. 37337030 PMC10397313

[37] S. A. Hussain , D. R. Ferry , G. El‐Gazzaz , et al., “Hepatocellular carcinoma,” Annals of Oncology: Official Journal of the European Society for Medical Oncology 12, no. 2 (2001): 161–172, 10.1023/a:1008370324827. 11300318

[38] Y. Yu and M. Feng , “Radiotherapy for Hepatocellular Carcinoma,” Seminars in Radiation Oncology 28, no. 4 (2018): 277–287, 10.1016/j.semradonc.2018.06.005. 30309638

[39] C. Corbet , E. Bastien , N. Draoui , et al., “Interruption of Lactate Uptake by Inhibiting Mitochondrial Pyruvate Transport Unravels Direct Antitumor and Radiosensitizing Effects,” Nature Communications 9, no. 1 (2018): 1208, 10.1038/s41467-018-03525-0. PMC5865202 29572438

[40] V. L. Payen , E. Mina , V. F. Van Hée , P. E. Porporato , and P. Sonveaux , “Monocarboxylate Transporters in Cancer,” Molecular Metabolism 33 (2020): 48–66, 10.1016/j.molmet.2019.07.006. 31395464 PMC7056923

[41] V. Pucino , M. Nefla , V. Gauthier , et al., “Differential Effect of Lactate on Synovial Fibroblast and Macrophage Effector Functions,” Frontiers in Immunology 14 (2023): 1183825, 10.3389/fimmu.2023.1183825. 37304267 PMC10251493

[42] Q. Tao , X. Li , T. Zhu , et al., “Lactate Transporter *SLC16A3* (MCT4) as an Onco‐Immunological Biomarker Associating Tumor Microenvironment and Immune Responses in Lung Cancer,” International Journal of General Medicine 15 (2022): 4465–4474, 10.2147/IJGM.S353592. 35509603 PMC9059363

[43] C. Wang , L. Xue , W. Zhu , L. Liu , S. Zhang , and D. Luo , “Lactate From Glycolysis Regulates Inflammatory Macrophage Polarization in Breast Cancer,” Cancer Immunology, Immunotherapy: CII 72, no. 6 (2023): 1917–1932, 10.1007/s00262-023-03382-x. 36729212 PMC10991532

[44] M. Certo , C. H. Tsai , V. Pucino , P. C. Ho , and C. Mauro , “Lactate Modulation of Immune Responses in Inflammatory Versus Tumour Microenvironments,” Nature Reviews Immunology 21, no. 3 (2021): 151–161, 10.1038/s41577-020-0406-2. 32839570

[45] A. D. Balgi , G. H. Diering , E. Donohue , et al., “Regulation of mTORC1 Signaling by pH,” PLoS One 6, no. 6 (2011): e21549, 10.1371/journal.pone.0021549. 21738705 PMC3126813

[46] A. El‐Kenawi , A. Ibrahim‐Hashim , K. Luddy , S. Pilon‐Thomas , R. Gatenby , and R. Gillies , “Abstract 3213: Extracellular Acidosis Alters Polarization of Macrophages,” Cancer Research 75 (2015): 3213, 10.1158/1538-7445.AM2015-3213.

[47] D. Xie , S. Zhu , and L. Bai , “Lactic Acid in Tumor Microenvironments Causes Dysfunction of NKT Cells by Interfering With mTOR Signaling,” Science China Life Sciences 59, no. 12 (2016): 1290–1296, 10.1007/s11427-016-0348-7. 27995420

[48] D. Langin , “Adipose Tissue Lipolysis Revisited (Again!): Lactate Involvement in Insulin Antilipolytic Action,” Cell Metabolism 11, no. 4 (2010): 242–243, 10.1016/j.cmet.2010.03.003. 20374952

[49] A. C. Scott , F. Dündar , P. Zumbo , et al., “TOX Is a Critical Regulator of Tumour‐Specific T Cell Differentiation,” Nature 571, no. 7764 (2019): 270–274, 10.1038/s41586-019-1324-y. 31207604 PMC7698992

[50] Y. Miao , J. Wang , Q. Li , et al., “Prognostic Value and Immunological Role of PDCD1 Gene in Pan‐Cancer,” International Immunopharmacology 89, no. Pt B (2020): 107080, 10.1016/j.intimp.2020.107080. 33069926

[51] J. Wen , X. Mao , Q. Cheng , Z. Liu , and F. Liu , “A Pan‐Cancer Analysis Revealing the Role of TIGIT in Tumor Microenvironment,” Scientific Reports 11, no. 1 (2021): 22502, 10.1038/s41598-021-01933-9. 34795387 PMC8602416

[52] A. Hosseini , T. Gharibi , F. Marofi , Z. Babaloo , and B. Baradaran , “CTLA‐4: From Mechanism to Autoimmune Therapy,” International Immunopharmacology 80 (2020): 106221, 10.1016/j.intimp.2020.106221. 32007707

[53] I. Siddiqui , K. Schaeuble , V. Chennupati , et al., “Intratumoral Tcf1(+)PD‐1(+)CD8(+) T Cells With Stem‐Like Properties Promote Tumor Control in Response to Vaccination and Checkpoint Blockade Immunotherapy,” Immunity 50, no. 1 (2019): 195–211.e10., 10.1016/j.immuni.2018.12.021. 30635237

