# Supplementary figures and images for: Integrated Single‐Cell and Spatial Transcriptome Reveal Metabolic Gene SLC16A3 as a Key Regulator of Immune Suppression in Hepatocellular Carcinoma
Source: J Cell Mol Med. 2024 Dec 10;28(23):e70272. doi: 10.1111/jcmm.70272 (PMC11629820; doi:10.1111/jcmm.70272)

A

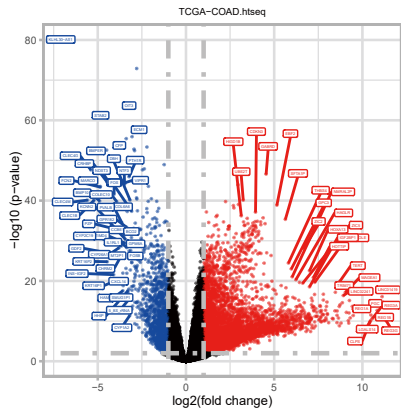

B

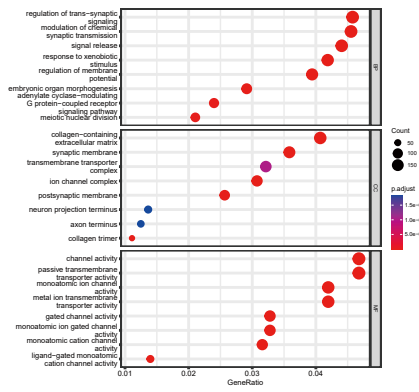

C

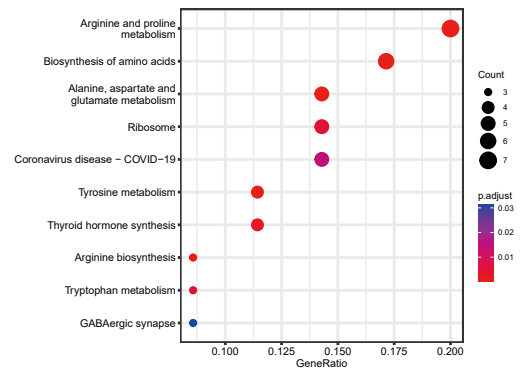

Supplement: Supplementary file 1 — Figure S1. Hepatocellular carcinoma public database transcriptome differential analysis. (A) Differential gene expression volcano plot. (B) Gene Ontology (GO) enrichment analysis of differentially expressed genes. (C) KEGG enrichment analysis of differentially expressed genes. [file JCMM-28-e70272-s004.pdf]

HCC1

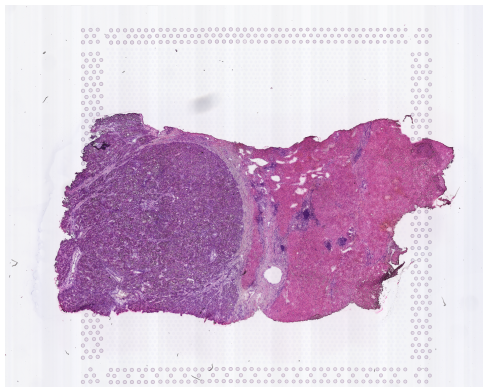

Clusters

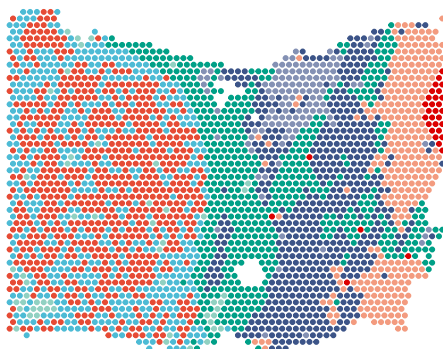

COL1A1

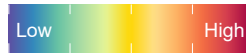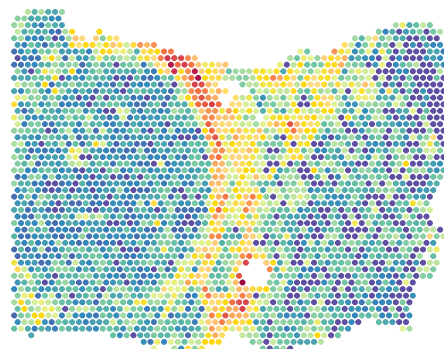

HCC3

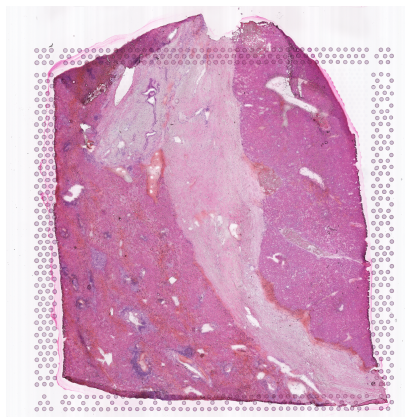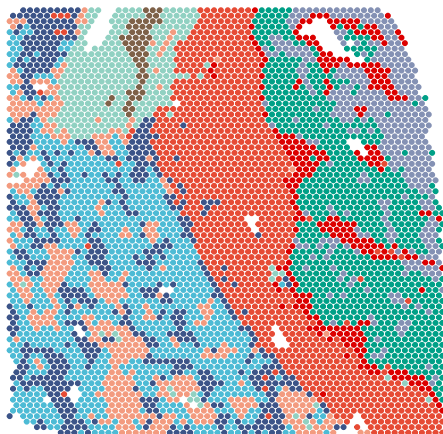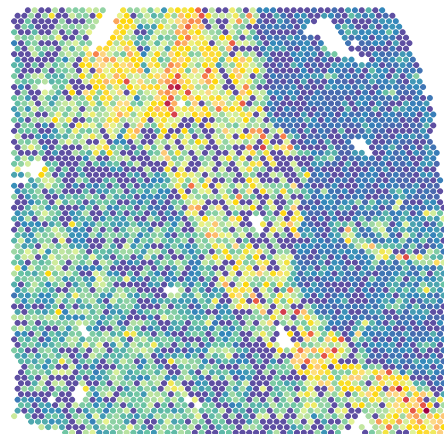

HCC4

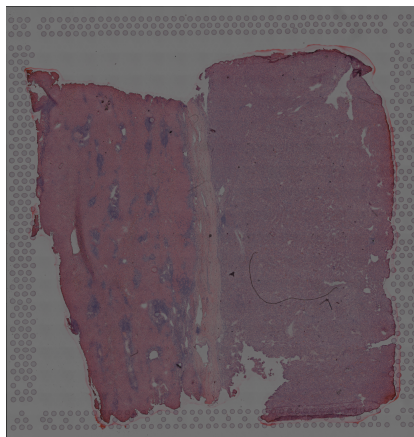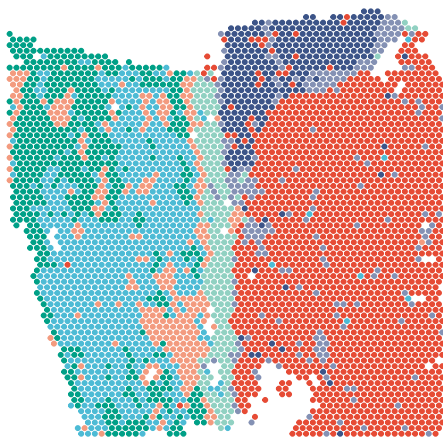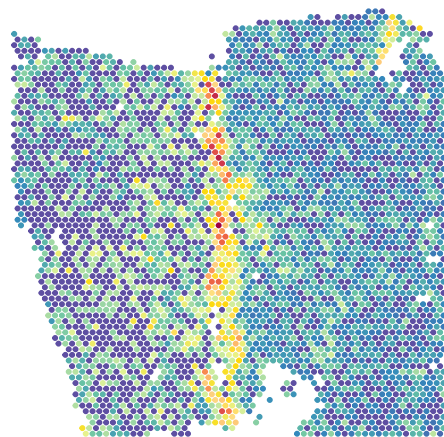

Supplement: Supplementary file 2 — Figure S2. Spatial transcriptome data of three samples. H&E staining (A), spatial cluster distribution (B) and spatial feature plots of COL1A1 expression (C) of each section. [file JCMM-28-e70272-s001.pdf]
